# Accumulation of epicardial fat rather than visceral fat is an independent risk factor for left ventricular diastolic dysfunction in patients undergoing peritoneal dialysis

**DOI:** 10.1186/1475-2840-12-127

**Published:** 2013-08-30

**Authors:** Heng-Hsu Lin, Jen-Kuang Lee, Chung-Yi Yang, Yu-Chung Lien, Jenq-Wen Huang, Cho-Kai Wu

**Affiliations:** 1 Division of Cardiology, Department of Internal Medicine, Far Eastern Memorial Hospital, New Taipei City, Taiwan; 2 Department of Laboratory Medicine, National Taiwan University Hospital, Taipei, Taiwan; 3 Department of Clinical Pathology & Cardiovascular Center, Far Eastern Memorial Hospital, New Taipei City, Taiwan; 4 Graduate Institute of Biomedical Electronics and Bioinformatics, National Taiwan University, Taipei, Taiwan; 5 Department of Medical Imaging, National Taiwan University College of Medicine and Hospital, Taipei, Taiwan; 6 Division of Nephrology, Department of Internal Medicine, National Taiwan University College of Medicine and Hospital, Taipei, Taiwan; 7 Division of Cardiology, Department of Internal Medicine, National Taiwan University College of Medicine and Hospital No. 7, Chung-Shan South Road Taipei 100, Taipei, Taiwan; 8 Graduate Institute of Clinical Medicine, College of Medicine, National Taiwan University, Taipei, Taiwan

**Keywords:** Diastolic dysfunction, Epicardial fat, Inflammation, Echocardiography

## Abstract

**Background:**

Symptoms of heart failure with preserved left ventricular systolic function are common among patients undergoing peritoneal dialysis (PD). Epicardial fat (EpF) is an ectopic fat depot with possible paracrine or mechanical effects on myocardial function. The aim of our current study is to assess the association between EpF and Left ventricular diastolic dysfunction (LVDD) in patients undergoing PD and to clarify the relationships among EpF, inflammation, and LVDD in this population.

**Methods:**

This was a cross-sectional study of 149 patients with preserved left ventricular systolic function who were undergoing PD. LVDD was diagnosed (according to the European Society of Cardiology guidelines) and EpF thickness measured by echocardiography. The patients without LVDD were used as controls. The serum inflammatory biomarker high-sensitivity C-reactive protein (hsCRP) was measured. The location and amount of adipose tissue were assessed by computed tomography (CT) at the level of the fourth lumbar vertebra.

**Results:**

Subjects with LVDD had higher levels of hsCRP, more visceral and peritoneal fat, and thicker EpF (all p < 0.001) than controls. Visceral adipose tissue, hsCRP, and EpF all correlated significantly (p < 0.05) with LVDD. Multivariate regression analysis rendered the relationship between visceral adipose tissue and LVDD insignificant, whereas EpF was the most powerful determinant of LVDD (odds ratio = 2.41, 95% confidence interval = 1.43–4.08, p < 0.01). EpF thickness also correlated significantly with the ratio of transmitral Doppler early filling velocity to tissue Doppler early diastolic mitral annular velocity (E/e’; r = 0.27, p < 0.01).

**Conclusion:**

EpF thickness is significantly independently associated with LVDD in patients undergoing PD and may be involved in its pathogenesis.

## Introduction

Cardiovascular disease (CVD) is the most common cause of mortality and morbidity among patients with end-stage renal disease (ESRD) undergoing either hemodialysis or peritoneal dialysis (PD) [1]. Patients with ESRD are prone to hypertension and left ventricular hypertrophy (LVH), which is a physiological response to pressure and volume overload. They also suffer disproportionately from fluid overload and systemic inflammation [2,3]. We recently found an interaction between PD and inflammation that further aggravates left ventricular diastolic dysfunction (LVDD) [4]. All of these contribute to the high prevalence of LVDD in patients undergoing PD [2,5]. Although LVDD could be an independent prognostic marker in patients with complex comorbidities [6], there, as yet, is scant information on the mechanisms of the pathogenesis of LVDD in patients undergoing PD.

Patients undergoing PD encounter complex nutritional problems. One of the most undesirable effects associated with PD is an increase in body fat mass [7,8]. Changes in body composition, including fat distribution, in patients undergoing PD are well described [9,10]. The distribution of body fat plays a pivotal role in the development and progression of both diastolic and systolic heart failure [11]. Epicardial fat (EpF) is the true visceral fat depot of the heart and accounts for approximately 20% of total heart weight [12]. There are currently several methods for evaluating EpF, including echocardiography, computed tomography (CT), and magnetic resonance image (MRI). Echocardiographic EpF measurement is the easiest method, and such measurements have been proven independently predictive of visceral adiposity and well correlated with myocardial fat [12,13]. EpF is a source of several proinflammatory and proatherogenic cytokines that influence cardiac function [13,14]. EpF also correlates with several cardiac comorbidities, including coronary artery disease (CAD) and left ventricular dysfunction [15-17]. However, there is as yet no evidence that the EpF thickness in patients receiving PD relates to LVDD.

Therefore, the present study was performed to assess the association between EpF and LVDD in patients undergoing PD and to clarify the relationships among EpF, inflammation, and LVDD in this population.

## Material and methods

### Study participants and study design

Between July 2007 and March 2009, 149 homogenous Taiwanese patients who had undergone PD using a conventional glucose-based lactate-buffered PD solution (UltraBag; Baxter Healthcare SA, Singapore) for >6 months at the specialty peritoneal dialysis clinic at the National Taiwan University Hospital were consecutively enrolled. Patients with hepatic disease, a history of myocardial infarction, coronary intervention, cardiac myopathy, or pericardial disease, chronic obstructive pulmonary disease, chronic atrial fibrillation, clinical signs of acute infection, or other chronic inflammatory conditions were excluded, as were patients taking statins, lipid-lowering agents, and/or other medication that could potentially influence relevant plasma parameters. All subjects underwent echocardiography and abdominal CT examination. In total, 65 participants were diagnosed with LVDD as defined by our previous reports and the recent consensus statement of the European Society of Cardiology. In brief, LVDD was defined echocardiographically by a ratio of early mitral valve flow velocity (E) to early diastolic lengthening velocity (e’; E/e’) during tissue Doppler imaging of ≥15 or by a combination of 15 > E/e’ ≥ 8, a mitral inflow E/A ratio < 0.5, and a deceleration time > 280 [18-20]. The remaining 84 subjects without LVDD served as the control group.

Written informed consent was obtained from every participating subject, and the study was approved by the institutional review board of the National Taiwan University Hospital.

### Biochemical data

Blood samples were collected from all patients from the antecubital vein between 8:00 AM and 10:00 AM with the patient in a supine position after fasting for 12 hr. No patient underwent PD exchange before sampling. Plasma glucose and serum total cholesterol (TC), high-density lipoprotein cholesterol (HDL), low-density lipoprotein cholesterol (LDL), triglycerides (TG), and high-sensitivity C-reactive protein (hsCRP) levels were determined using an automatic analyzer (Toshiba TBA 120FR, Toshiba Medical Systems Co., Ltd., Tokyo, Japan). All samples were processed blindly by examiners who were unaware of the patients’ clinical characteristics.

### Echocardiography and measurement of EpF

**Figure 1:**
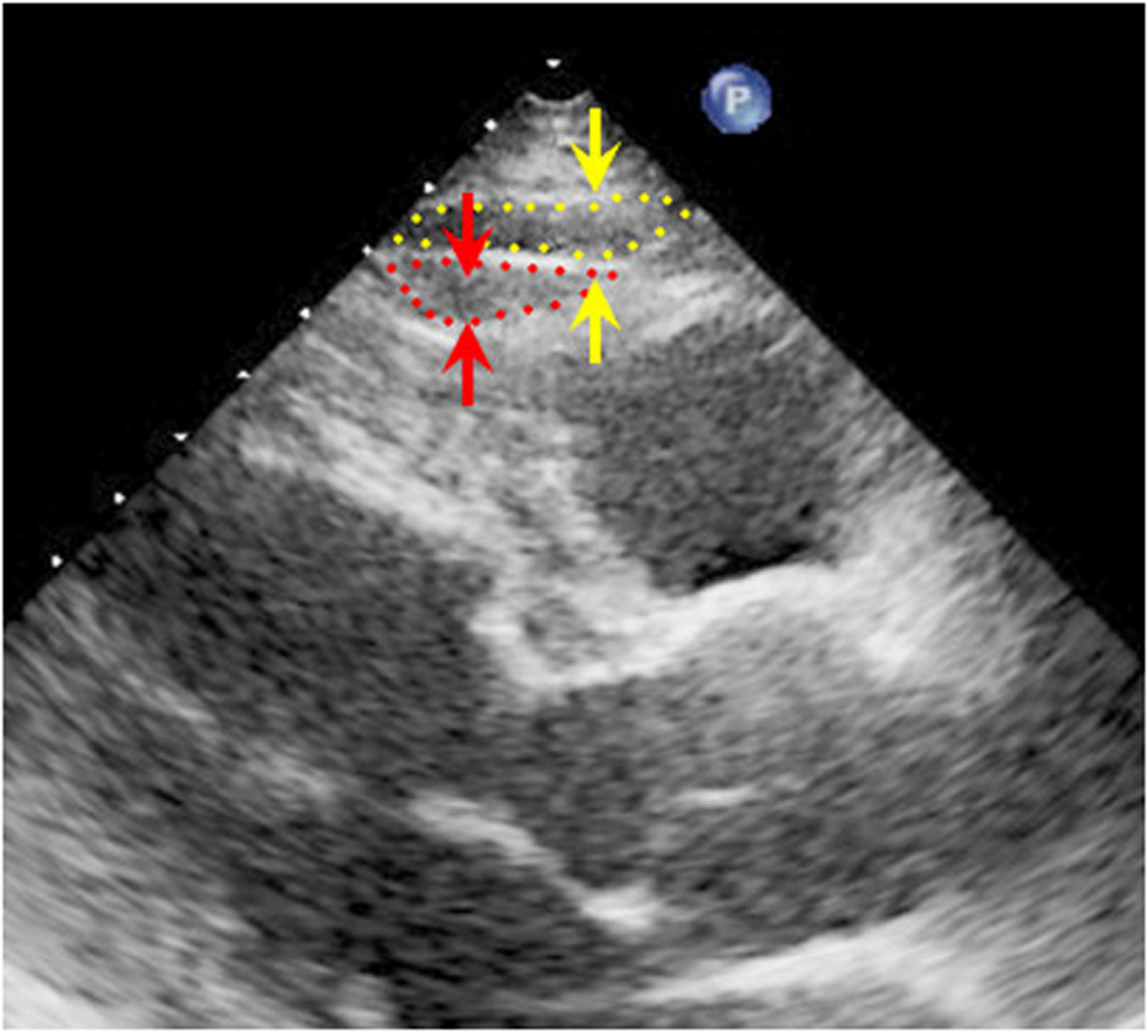
**Echocardiographic images of a 55-year-old male patient with hypertension, dyslipidemia, and diabetes.** The red area is the epicardial fat within the pericardial sac as quantified 3-dimensionally by echocardiography.

Left atrial (LA) diameter, the LV end-diastolic and systolic diameters, interventricular septum thickness, LV posterior wall thickness, mitral inflow early rapid filling wave (E), peak velocity of the late filling wave due to atrial contraction (A), E/A ratio, E wave deceleration time, and mitral annular early diastolic velocity were measured according to the American Society of Echocardiography guidelines using an iE33 xMATRIX echocardiography system (Philips Healthcare, Best, the Netherlands). The LV mass index was calculated from the LV end-diastolic and systolic diameters, interventricular septum thickness, and LV posterior wall thickness according to the method of Devereux et al. [21]. The peak annular early and late diastolic velocities of the lateral mitral annulus during tissue Doppler imaging (e’ and a’) were also recorded. Doppler and color Doppler studies were performed to detect valvular heart disease. Significant valvular heart disease was defined as at least moderate aortic or mitral stenosis/regurgitation. EpF was measured by 2 readers with no knowledge of the patients’ baseline characteristics according to previously published methods [22]. In brief, EpF was identified as the echocardiographically free space between the outer wall of the myocardium and the visceral layer of the pericardium, and its thickness was measured perpendicularly to the free wall of the right ventricle at end-systole over 3 cardiac cycles (Figure 1). The mean value of 3 cardiac cycles from each echocardiographic view (including both parasternal long- and short-axis views) was recorded as the EpF thickness. Repeat analysis of 20 patients was performed by either the same or a second observer. Inter-observer correlation coefficients were 0.89 whereas intra-observer correlation coefficients were 0.93.

### Determination of adipose tissue distribution by CT

Each subject was imaged using a 16-MDCT scanner (LightSpeed 16, GE Healthcare, Milwaukee, WI, USA). The images were analyzed using image analysis software (ImageJ, version 1.43q; National Institutes of Health, Bethesda, MD, USA) with an attenuation range of −50 to −250 Hounsfield units to quantify the subcutaneous, visceral, and total abdominal adipose tissue areas at the level of the umbilicus. The results were expressed in centimeter square. The visceral/subcutaneous adipose tissue area ratio (V/S ratio) was calculated.

### Statistical analysis

Analyses were performed using SPSS 16.0 for Windows XP (SPSS, Inc., Chicago, IL, USA). The Kolmogorov-Smirnov test was used to determine whether the data were normally distributed. Data for continuous variables that were not normally distributed were transformed using the Napierian logarithm. Baseline characteristics and echocardiographic findings were compared between groups using Student’s unpaired *t* test for continuous data and a chi-squared test for categorical data. Comparisons of mean values across groups and correlations between continuous variables were assessed via linear regression. Odds ratios (ORs) and 95% confidence intervals (CIs) were estimated by logistic regression. Associations between EpF and echocardiographic diastolic function parameters were determined using Pearson’s correlation coefficient. Multivariate models were used to assess the associations of adipose tissue and serum biomarkers with LVDD. Covariates associated with LVDD, including age, diabetes, hypertension, and the natural logarithms of the LDL level and left ventricular mass index, were incorporated. All p values were 2-sided, and p values < 0.05 were considered indicative of statistical significance.

## Results

### Demographics

Of the 149 subjects in our sample population, 65 were diagnosed with LVDD, and the remainder served as controls. The baseline characteristics of the participants in both groups are summarized in Table 1. Consistent with previous reports on hypertension, the subjects with LVDD were predominantly female, older, and suffered more frequently from hypertension or hyperlipidemia (higher LDL level). Patients with LVDD had significantly higher hsCRP levels (Table 1). The cause of renal failure and the residual renal function did not differ between the groups. Relative to the control group, subjects with LVDD had larger end-diastolic and systolic LV volumes (p < 0.05), greater LA diameters, and larger indexed LV mass values (p < 0.05). Comparison of the functional parameters showed a prolonged deceleration time (DT) (p < 0.05), increased mitral inflow late filling wave (p < 0.001), decreased mitral inflow E/A ratio (p < 0.005), and decreased peak annular early diastolic velocity of the lateral mitral annulus in tissue Doppler imaging (p < 0.001) among the patients with LVDD.

**Table 1:** Baseline demographic data, echocardiographic characteristics, and adipose tissue measurements of the 149 patients undergoing peritoneal dialysis included in the study

	**No LVDD**	**LVDD**	
**Baseline characteristics**	**(n = 84)**	**(n = 65)**	**p**
Age (years)	53.4 ± 14.8	57.8 ± 11.7	0.035*
Women (%)	43 (51)	40 (62)	0.25
HTN (n)	50	56	<0.001*
DM (n)	23	25	0.16
Fasting plasma glucose (mg/dL)	96.2 ± 19.5	97.3 ± 11.8	0.69
HDL (mg/dL)	38.9 ± 8.3	41.7 ± 12.6	0.26
LDL (mg/dL)	83.2 ± 33.5	99.1 ± 38.0	0.05*
TG (mg/dL)	125.3 ± 85.3	142.4 ± 96.1	0.23
Height (cm)	163 ± 5.9	162 ± 6.5	0.30
Body weight (kg)	59 ± 10.5	60 ± 11.7	0.82
BMI (kg/m^2^)	23 ± 3.9	24 ± 3.2	0.46
CRP (mg/dL)	0.74 ± 0.99	2.13 ± 1.73	<0.001*
Primary renal diagnosis			
DM	29	25	0.61
CGN	25	17	0.59
Other	30	23	0.87
Time on PD (h)	18.9 ± 1.6	19.1 ± 1.3	0.46
Kt/V	1.72 ± 0.34	1.91 ± 0.25	0.57
Residual renal function (mL/min)	1.94 ± 0.31	2.31 ± 0.22	0.25
**Echocardiographic characteristics**			
LV end-diastolic diameter (mm)	48.4 ± 6.7	50.9 ± 7.4	0.03*
LV end-systolic diameter (mm)	30.4 ± 6.2	32.5 ± 8.1	0.04*
LA diameter (mm)	32.6 ± 6.5	37.3 ± 5.8	<0.001*
LV ejection fraction (%)	64.4 ± 7.9	66.2 ± 9.1	0.17
E (cm/s)	71.4 ± 20.0	89.4 ± 32.0	<0.001*
A (cm/s)	88.6 ± 20.2	104.8 ± 30.1	<0.001*
E/A	0.97 ± 0.46	0.77 ± 0.35	0.003*
DT (ms)	200.9 ± 45.2	238.7 ± 78.7	<0.001*
e’ (cm/s)	7.6 ± 2.4	5.7 ± 2.0	<0.001*
E/e’	10.2 ± 4.0	17.0 ± 8.1	<0.001*
LV mass index (g/cm^3^)	213.4 ± 63.9	245.3 ± 77.6	0.03*
EpF (mm)	2.8 ± 1.6	5.1 ± 2.6	<0.001*
**Adipose tissue distribution**			
Total fat (cm^2^)	242.7 ± 87.2	335.0 ± 207.5	0.001*
Subcutaneous fat (cm^2^)	123.8 ± 60.6	170.2 ± 79.3	0.001*
Visceral fat (cm^2^)	89.3 ± 60.4	128.1 ± 77.1	0.006*
Peritoneal fat (cm^2^)	52.2 ± 45.9	88.5 ± 71.2	0.003*
Retroperitoneal fat (cm^2^)	385.0 ± 241.5	481.3 ± 289.1	0.07

The data are presented as the mean ± standard deviation unless otherwise indicated.

*Abbreviations:LVDD* left ventricular diastolic dysfunction; *BMI* body mass index; *WC* waist circumference; *DM* diabetes mellitus; *CGN* chronic glomerular nephritis; *HTN* hypertension; *HOMA* homeostasis model of insulin resistance; *CRP* C-reactive protein; *LDL* low density lipoprotein; *HDL* high density lipoprotein; *TG* triglyceride; *PD* peritoneal dialysis; kt/v, k = dialyzer clearance of urea, t = dialysis time, v = volume of distribution of urea, approximately equal to the patient’s total body water; *LA* left atrial; *LV* left ventricular; *E* mitral inflow E wave; *A* mitral inflow A wave; *DT* mitral inflow deceleration time; e’, peak annular early diastolic velocity of the lateral mitral annulus in tissue Doppler imaging; *IVRT* interventricular relaxation time; *EpF* epicardial fat. *, p < 0.05.

Comparison of the anthropometric characteristics showed higher levels of markers reflecting fat distribution, such as the amounts of total, subcutaneous, visceral, and peritoneal fat (p < 0.001, p < 0.001, p < 0.01, and p < 0.005, respectively), in the LVDD group.

### Correlation between EpF thickness and LVDD

**Figure 2:**
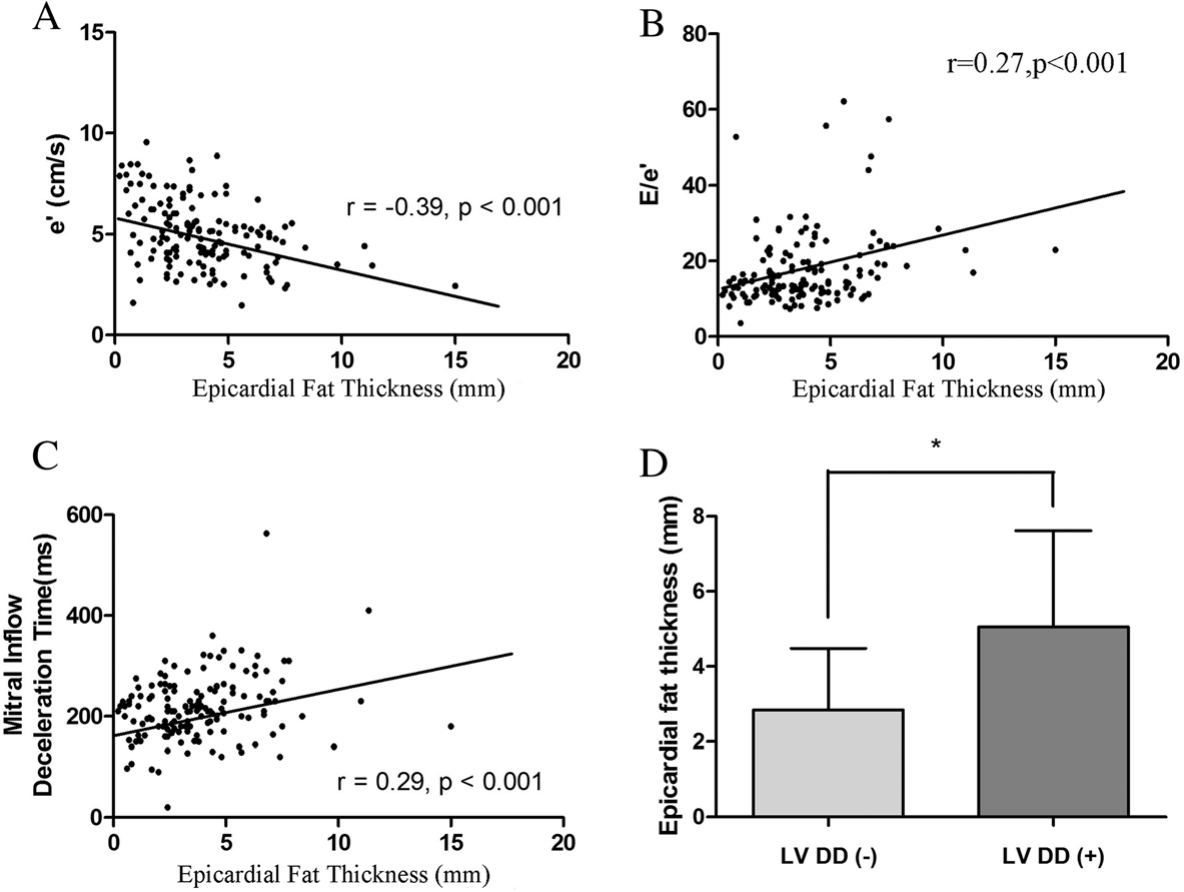
**Correlation between epicardial fat (EpF) thickness and left ventricular diastolic dysfunction (LVDD).** EpF thickness correlated significantly with **(A)** e’ (r = −0.39, p < 0.001), **(B)** E/e’ (r = 0.27, p < 0.001), and **(C)** mitral inflow deceleration time (r = 0.29, p = 0.002). **(D)** EpF thickness in patients with or without left ventricular diastolic dysfunction (LVDD). The bar graph shows the mean + standard deviation. EpF thickness was significantly greater in patients with LVDD (5.1 ± 2.6 cm^3^) than in controls (2.8 ± 1.6 cm^3^, p < 0.001).

The bivariate Pearson correlation coefficients for LV diastolic function parameters and EpF are shown in Figure 2. EpF was significantly associated with tissue Doppler e’, E/e’, and DT (r = −0.39, p < 0.001; r = 0.27, p = 0.001; r = 0.29, p < 0.001, respectively) (Figure 2A–C). EpF thickness was greater in patients with LVDD (n = 65; 5.1 ± 2.6 mm) than in controls (n = 84; 2.8 ± 1.6 mm, p < 0.001, Figure 2D).

### Factors associated with LVDD

We performed univariate analysis to determine the risk factors associated with the development of LVDD. Hypertension, the natural logarithm of the LDL level [Ln(LDL)], Ln(Visceral fat), Ln(Peritoneal fat), the hsCRP level, Ln(LV mass index) and EpF thickness were significantly associated with the presence of LVDD (Table 2).

**Table 2:** Univariate logistic regression analyses of risk factors associated with the presence of LVDD

	**OR (95% CI)**	**p**
Age	1.02 (0.99–1.04)	0.14
Gender	1.53 (0.80–2.94)	0.21
BMI	1.05 (0.92–1.20)	0.45
DM	1.66 (0.83–3.31)	0.15
Hypertension	4.11 (1.80–9.42)	<0.001
Ln(LDL)	3.38 (1.01–11.36)	0.049
Ln(TG)	0.95 (0.48–1.88)	0.88
Ln(Visceral fat)	2.09 (1.18–3.68)	0.01
Ln(Peritoneal fat)	1.92 (1.22–3.02)	0.005
hsCRP	2.09 (1.56–2.79)	<0.001
Ln(LV mass index)	4.98 (1.10–22.64)	0.03
EpF	1.75 (1.41–2.17)	<0.001

*Abbreviations:LVDD,* left ventricular diastolic dysfunction; *OR,* odds ratio; *CI,* confidence interval; *BMI,* body mass index; *DM,* diabetes mellitus; *Ln*(LDL), natural logarithm of low-density lipoprotein; *TG,* triglyceride; *hsCRP,* high-sensitivity C-reactive protein; *LV,* left ventricular; *EpF,* epicardial fat. ORs and 95% CIs were estimated by logistic regression.

### Accumulation of EpF rather than visceral fat is an independent risk factor for LVDD

We previously found subclinical inflammation (e.g., the plasma hsCRP level) to be an independent risk factor for LVDD in patients with metabolic syndromes [4]. We therefore performed multivariate analysis of the risk factors other than adiposity that were associated with LVDD in our univariate analyses and found that the hsCRP level remained associated with LVDD after adjustment for age, gender, diabetes mellitus, hypertension, the hsCRP and LDL levels, and the LV mass index (OR: 1.75, 95% CI: 1.08–3.12, p = 0.038; Table 3, model 1).

**Table 3:** Multivariate logistic regression models for parameters associated with left ventricular diastolic dysfunction

	**Odds ratio**	**95% Confidence interval**	**p**
Model 1			
Age	1.07	1.01–1.14	0.018*
Ln(LV mass index)	14.88	1.91–115.7	0.01*
DM	1.60	0 50–5.12	0.425
HTN	2.18	0.48–9.93	0.313
Ln(LDL)	5.54	1.31–23.52	0.02*
hsCRP	1.75	1.08–3.12	0.038*
Model 2			
Ln(Visceral Fat)	2.29	1.06–5.57	0.013*
Model 3			
Ln(Peritoneal Fat)	2.45	1.1–5.92	0.011*
Model 4			
Ln(Visceral Fat)	2.10	0.85–6.43	0.344
hsCRP	2.56	1.08–4.72	0.023*
Model 5			
Ln(Peritoneal Fat)	2.23	0.85–6.78	0.268
hsCRP	2.23	1.21–6.69	0.019*
Model 6			
EpF	2.41	1.43–4.08	0.01*
Ln(visceral Fat)	2.04	0.48–6.68	0.14
Peritoneal Fat	3.15	0.12–8.65	0.50
hsCRP	3.03	1.26–7.30	0.01*

*Abbreviations:Ln(LV mass index),* natural logarithm of the left ventricular mass index; *DM,* diabetes mellitus; *HTN,* hypertension; *hsCRP,* high-sensitivity C-reactive protein; *LDL,* low-density lipoprotein; *EpF,* epicardial fat. Odds ratios and 95% confidence intervals were estimated by logistic regression. Models 2–6 adjust for age, DM, HTN, Ln(LDL), and Ln(LV mass index). Model 4 and model 5 additionally adjust for hsCRP. Model 6 additionally adjusts for hsCRP and adiposity components. *, p < 0.05.

We further evaluated fat distribution and its association with LVDD by multivariate regression analysis. Of the CT measures of fat distribution, Ln(visceral fat) and Ln(peritoneal fat) remained associated with LVDD after adjustment for the abovementioned confounding factors (adjusted OR = 2.29, 95% CI = 1.06–5.57, p = 0.013 and adjusted OR = 2.45, 95% CI = 1.1–5.9, p = 0.011 respectively; Table 3, models 2–3). We repeated the multivariate analysis with adjustment for the hsCRP level and found that this abolished the associations of visceral and peritoneal fat with LVDD. The effect of the hsCRP level itself remained significant in both models (OR = 2.56, 95% CI = 1.08–4.72, p = 0.023 and OR = 2.70, 95% CI = 1.21–6.69, p = 0.019, respectively; Table 3, model 4 and model 5), indicating that hsCRP might mediate the effect of visceral and/or peritoneal fat on LVDD. To elucidate the influence of EpF, we adjusted for all of the confounding factors and parameters of inflammation and adiposity simultaneously in model 6. EpF remained an independent, significant predictor of the presence of LVDD in this model, which may imply that EpF influences LV diastolic function through pathways other than subclinical inflammation.

## Discussion

In this study, we found that EpF thickness in patients undergoing PD patients correlates with LVDD even after adjustment for all of the confounding factors, whereas visceral fat was not significantly independently associated with LVDD. In addition, LVDD was significantly associated with the hsCRP level in this specific group of patients. Inflammation and EpF were the 2 most important risk factors for LVDD, implying that these factors might act synergistically.

Although there is no published figure for the prevalence of diastolic heart failure in patients undergoing PD, LV diastolic heart failure would be expected more frequently among patients with ESRD than in the general population due to the inflammation, fluid overload, hypertension, renin-angiotensin-aldosterone system activation, and LV hypertrophy associated with ESRD [2,23]. Abnormal fat distribution seems a likely culprit for the high prevalence of LVDD in patients undergoing PD. Such patients tend to have elevated amounts of intra-abdominal fat [24], and we recently found higher amounts of visceral adipose tissue to be associated with low-grade inflammation, which leads in turn to subclinical LVDD [4]. However, the current study is to the best of our knowledge the first report that inflammation and EpF content are independent risk factors for LVDD in patients with PD and thus suggests some explanations for the prevalence of LVDD in this group. EpF is characterized by a high rate of release of free fatty acids (FFA) [25], which encounter no physical barrier or fascia before reaching the cardiomyocytes [26]; therefore, the myocardium receives a double dose of FFA from both the EpF and the systemic circulation.

There are also hypothesis that EpF can influence LV diastolic function. EpF is a source of several bioactive molecules that might directly influence the myocardium [27]. In metabolic and cardiovascular disease states, these fat tissues expand, becoming hypoxic and dysfunctional [28,29] and recruiting phagocytic cells [30] which would lead to reducing the production of protective cytokines, increasing detrimental adipocytokines and impaired cardiac function eventually. Besides, Iozzo P et al. have documented that the entire mass of fat surrounding the heart ranges on average from 100 g (healthy individual) to 400 g (type II DM patient), extending to 800 ~ 900 g in some patients [31]. This weight probably poses a mechanical burden on cardiac expansion, and may further deteriorate LVDD in PD patients. The diagram for the above discussion is summarized in Additional file 1.

Increased levels of myocardial triglycerides could cause cardiomyocyte apoptosis, increased oxidative stress, and impairment of cardiac function. Kankaanpää et al. evaluated EpF and left ventricular (LV) function using MRI and concluded that FFA exposure and EpF may increase accumulation of myocardial triglycerides, which might have negative effect for LV overload and hypertrophy [32]. A recent study assessing the association between echocardiographic EpF and myocardial fat found that echocardiographic EpF accumulation might reflect myocardial fat in subjects with a wide range of adiposity [22]. Taken together, the current study and previous evidence suggest that the mechanisms delineated in animal models also operate in patients undergoing PD. These patients have abnormal fat distribution, and accumulation of fat, especially visceral fat, can potently exacerbate inflammation. In addition, recent studies show that epicardial adipose tissue plays an important role of development and progression of CAD. Elevated inflammatory infiltrate has been described in EpF of subjects with CAD [33]. When compared with subcutaneous fat, inflammatory cell infiltration, mainly contributed by macrophages, is enhanced in epicardial adipose tissue [33,34]. Besides of macrophage accumulation, macrophage phenotypic change in epicardial adipose tissue of CAD patients is also demonstrated [35]. Moreover, as we look into the role of visceral fat in the pathogenesis of CAD, there is evidence which suggests that not only EpF but mediastinal adipose tissue contributes locally to the development of coronary atherosclerosis via glucocorticoid action [36]. Increased epicardial fat thickness is shown to be an independent risk factor for CAD in specific patients [37], and there are probably gender disparities in the association between epicardial adipose tissue volume and CAD [38].

In our current study, we measured the amount of EpF by echocardiography. Malavazos et al. [22] documented that echocardiographic measurement accurately estimates the actual amount of EpF, which could be a strong and independent predictor of myocardial fat (represented by triglycerides stored within the cardiac muscle tissue). Echocardiographic EpF measurement has advantages for use in both clinical and research settings, including low cost, routine applicability, avoidance of exposure to radiation, and potential for monitoring therapeutic effects. As myocardial fat has progressive and harmful effects on LV diastolic function, accumulation of EpF may affect the heart as well [39]. Atrial enlargement has been shown to correlate with impairment in diastolic filling in morbidly obese subjects [40]. In our current study, we controlled for the influence of systemic adiposity and its effect via systemic inflammation and still found an independent role for the amount of EpF in the development of LVDD. These results not only reveal that EpF directly reflects myocardial fat content but also allow us to monitor early myocardial changes in patients undergoing PD. This convenient method might even be able to detect the development of LVDD, allowing early initiation of treatment and monitoring of the response of therapy, which is especially important in patients with multiple co-morbidities.

An elevated or excessive amount of adipose tissue is regarded as a risk factor for a number of diseases, including insulin resistance, type 2 diabetes mellitus, and atherosclerosis, all of which can lead to major cardiovascular sequelae. Further, adipose tissue is associated with changes in both inflammatory cells and biochemical markers of inflammation [23,41]. In the current study, we demonstrated that inflammation and accumulation of EpF play pivotal roles in the development of LVDD in patients undergoing PD. However, the associations therewith of visceral and peritoneal adipose tissue were no longer significant after adjustment for inflammation. We recently assessed the role of pro-inflammatory cytokines in the relationship between inflammation and LVDD in patients undergoing PD [42]. That study identified a significant correlation between LVDD and serum markers of inflammation in patients undergoing PD and an interaction between PD and inflammation. Taking these results together with the findings of the current study, we speculate that the systemic load of adipose tissue in patients undergoing PD might increase the susceptibility to LVDD through inflammation, while EpF represents the true amount of myocardial fat and thus acts independently. Inflammation (contributed in part by systemic adipose tissue) and increase of triglycerides stored within the cardiac muscle tissue (proportional to EpF) could therefore act synergistically to cause LVDD.

Our study had several limitations. First, we used cross-sectional data to infer longitudinal relationships. Although a model was identified, additional associations and possible pathways may exist, and further, prospective studies will be necessary to delineate a precise causal relationship. Second, all of the patients in our current study are Taiwanese. The generalizability to other population should be demonstrated by further study. Third, the effects of LVDD and EpF thickness on the long-term outcomes of patients undergoing PD warrant further investigation.

In conclusion, we delineated for the first time the complex relationships among EpF accumulation, inflammation, and LVDD in patients undergoing PD. This study demonstrated that the amount of EpF but not visceral adipose tissue is independently associated with subclinical LVDD in patients undergoing PD.

## Abbreviations

CVD: Cardiovascular disease; ESRD: End-stage renal disease; PD: Peritoneal dialysis; LVDD: Left ventricular diastolic dysfunction; EpF: Epicardial fat; CAD: Coronary artery disease; HSCRP: High sensitivity c-reactive protein; BMI: Body mass index; DM: Diabetes mellitus; HOMA: Homeostasis model of insulin resistance; LDL: Low density lipoprotein; HDL: High density lipoprotein; TG: Triglyceride; E: Mitral inflow E wave; A: Mitral inflow A wave; DT: Mitral inflow deceleration time; e’: Peak annular early diastolic velocity of the lateral mitral annulus in tissue doppler imaging.

## Competing interests

The corresponding author has full access to all of the data in the study and takes responsibility for the integrity of the data and the accuracy of the data analysis. The authors declare that they have no competing interests.

## Authors’ contributions

Regarding the contribution of each author, CW designed the whole study. CY and JL analyzed and interpreted the data. YL, HL, CW performed the laboratory work, and wrote the manuscript. CW, JH recruited the patients, and critically reviewed the manuscript for important intellectual content. CW was also in charge of the whole program. All authors read and approved the final manuscript.

## Supplementary Material

Additional file 1 The diagram for the hypothesis of how epicardial fat influence left ventircular diastolic function. Click here for file
