# Supplementary figures and images for: Accumulation of epicardial fat rather than visceral fat is an independent risk factor for left ventricular diastolic dysfunction in patients undergoing peritoneal dialysis
Source: Cardiovasc Diabetol. 2013 Aug 30;12:127. doi: 10.1186/1475-2840-12-127 (PMC3766214; doi:10.1186/1475-2840-12-127)

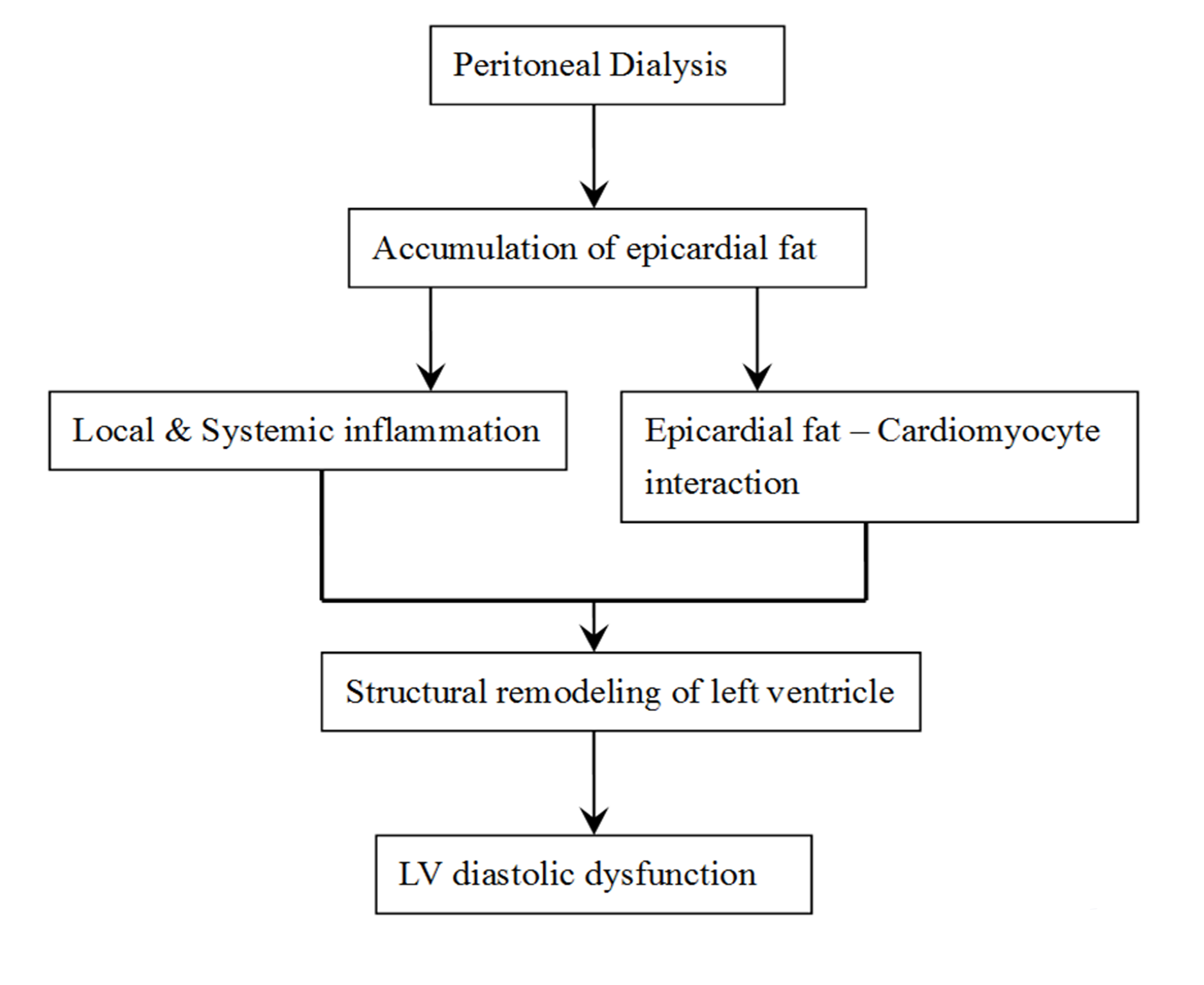

Supplement: Additional file 1 — The diagram for the hypothesis of how epicardial fat influence left ventircular diastolic function. [file 1475-2840-12-127-S1.tiff]
